# Altered Microbiota, Impaired Quality of Life, Malabsorption, Infection, and Inflammation in CVID Patients With Diarrhoea

**DOI:** 10.3389/fimmu.2020.01654

**Published:** 2020-07-31

**Authors:** Cornelia M. van Schewick, Christina Nöltner, Svenja Abel, Siobhan O. Burns, Sarita Workman, Andrew Symes, David Guzman, Michele Proietti, Alla Bulashevska, Fernando Moreira, Veronika Soetedjo, David M. Lowe, Bodo Grimbacher

**Affiliations:** ^1^Institute of Immunity and Transplantation, Royal Free Hospital, University College London, London, United Kingdom; ^2^Center for Chronic Immunodeficiency, Medical Center, Faculty of Medicine, Institute for Immunodeficiency, Albert-Ludwigs-University Freiburg, Freiburg, Germany; ^3^Freiburg Center for Data Analysis and Modeling (FDM), IMBI/ZKS, Freiburg, Germany; ^4^DZIF – German Center for Infection Research, Satellite Center Freiburg, Freiburg, Germany; ^5^CIBSS - Centre for Integrative Biological Signalling Studies, Albert-Ludwigs-University Freiburg, Freiburg, Germany; ^6^RESIST – Cluster of Excellence 2155 to Hanover Medical School, Satellite Center Freiburg, Freiburg, Germany

**Keywords:** CVID, diarrhoea, calprotectin, norovirus, microbiome, IBDQ, lymphocytes, quality of life

## Abstract

**Background:** Diarrhoea is the commonest gastrointestinal symptom in patients with common variable immunodeficiency (CVID).

**Objective:** The aim of this study was to describe the prevalence and clinical presentation of chronic and recurrent diarrhoea in the Royal-Free-Hospital (RFH) London CVID cohort, including symptoms, infections, level of inflammation, and microbial diversity.

**Methods:** A cross-sectional study of adult CVID patients (139 out of 172 diagnosed with CVID completed the screening questionnaire). Those with diarrhoea ≥6 days/month had stool and blood samples analysed and completed the short Inflammatory Bowel Disease Questionnaire (sIBDQ). BMI, spleen-size, lymphocytes and gut-microbial diversity were compared. Due to logistical and clinical restraints, not all patients could be analysed on all measures.

**Results:** 46/139 (33.1%) patients had current significant diarrhoea. In patients with past or present diarrhoea, BMI was lower (median 23.7 vs. 26, *p* = 0.005), malabsorption more common (57.97 vs. 35.71%, *p* = 0.011). CD4+ lymphocytes were higher in patients with diarrhoea (*p* = 0.028; *n* = 138), but CD4+ naïve lymphocytes were significantly higher in non-diarrhoea patients (*p* = 0.009, *N* = 28). Nine patients had confirmed or probable current gastrointestinal infections. Calprotectin was >60 μg/g in 13/29 with significant diarrhoea including 9 without infection. SIBDQ revealed a low median score of 4.74. Microbial alpha diversity was significantly lower in CVID patients compared to healthy household controls. There was no significant difference in alpha diversity in relation to antibiotic intake during the 6 weeks prior to providing samples.

**Conclusion:** Patients with CVID and significant diarrhoea had infections, raised calprotectin, malabsorption, a lower BMI, an impaired quality of life (comparable to active IBD), and they differed from non-diarrhoea patients in their lymphocyte phenotyping. Furthermore, microbial diversity was altered. These findings strongly imply that there may be an inflammatory nature and a systemic predisposition to diarrhoea in CVID, which necessitates further investigation.

## Introduction

Common variable immunodeficiency (CVID) is characterised by very low immunoglobulin levels of IgG and IgA or IgM (1) with a poor vaccination response due to impaired B-cell function, raised susceptibility toward infections, and autoimmune, or granulomatous manifestations. It is the commonest symptomatic primary immunodeficiency (PID) in humans (2), representing a heterogeneous set of disorders (3).

Abdominal symptoms are a burden to a large part of investigated CVID cohorts (4–8). Their reported prevalence varies from 21 to 47% (4, 8). For chronic diarrhoea, defined as diarrhoea for ≥4 weeks with or without a minimum of three bowel movements per day (9), numbers vary between 7 and 14% at time of diagnosis (7, 8) and between 22 and 77% during follow up (6–8). This makes diarrhoea the commonest abdominal symptom in CVID patients.

Immunodeficient patients may have acute or chronic *Giardia* spp. Infections (8, 10) and they can have chronic norovirus infections (11, 12) where bowel histology resembles coeliac disease and faecal calprotectin levels can be raised (13, 14). *Campylobacter* infections may also occur in CVID patients, including as a chronic infection (8, 15).

However, in addition to chronic infection, there is the suggestion that many CVID bowel complications are a separate entity, or several disease entities, which do not fit common classifications (16). The term “CVID enteropathy,” although it lacks a clear working definition, has been used to describe diverse pathological entities. We therefore focussed on the symptom of diarrhoea and sought to further understand its impact and associations.

In order to assess the relevance of “diarrhoea,” surrogate markers can be used. Calprotectin is a widely used marker indicative of (especially neutrophilic) inflammation (17). The short Inflammatory Bowel Disease Questionnaire (sIBDQ) is a validated 10 item questionnaire for the assessment of the quality of life in IBD patients (18, 19), which may be applicable to other diseases.

A system to classify the heterogenous group of CVID patients is the Euroclass classification system (20), which defines B-lymphocyte dependent phenotypes correlating with clinical features.

So far, only few investigations of the CVID microbiome have been published (21–26). The diversity of microbiota within one individual or its sample (alpha diversity) and the differences in distribution of different microbiota among samples or individuals (beta diversity) can be described. The microbiota reflects many influencing factors such as diet (27, 28), antibiotic intake (29), and genetics (30). The microbiome varies greatly interindividually (31).

The aim of this study was to describe the prevalence and clinical presentation of chronic and recurrent diarrhoea in the Royal-Free-Hospital (RFH) London CVID cohort, including symptoms, infections, level of inflammation, and the gut microbiota.

## Materials and Methods

**Figure 1:**
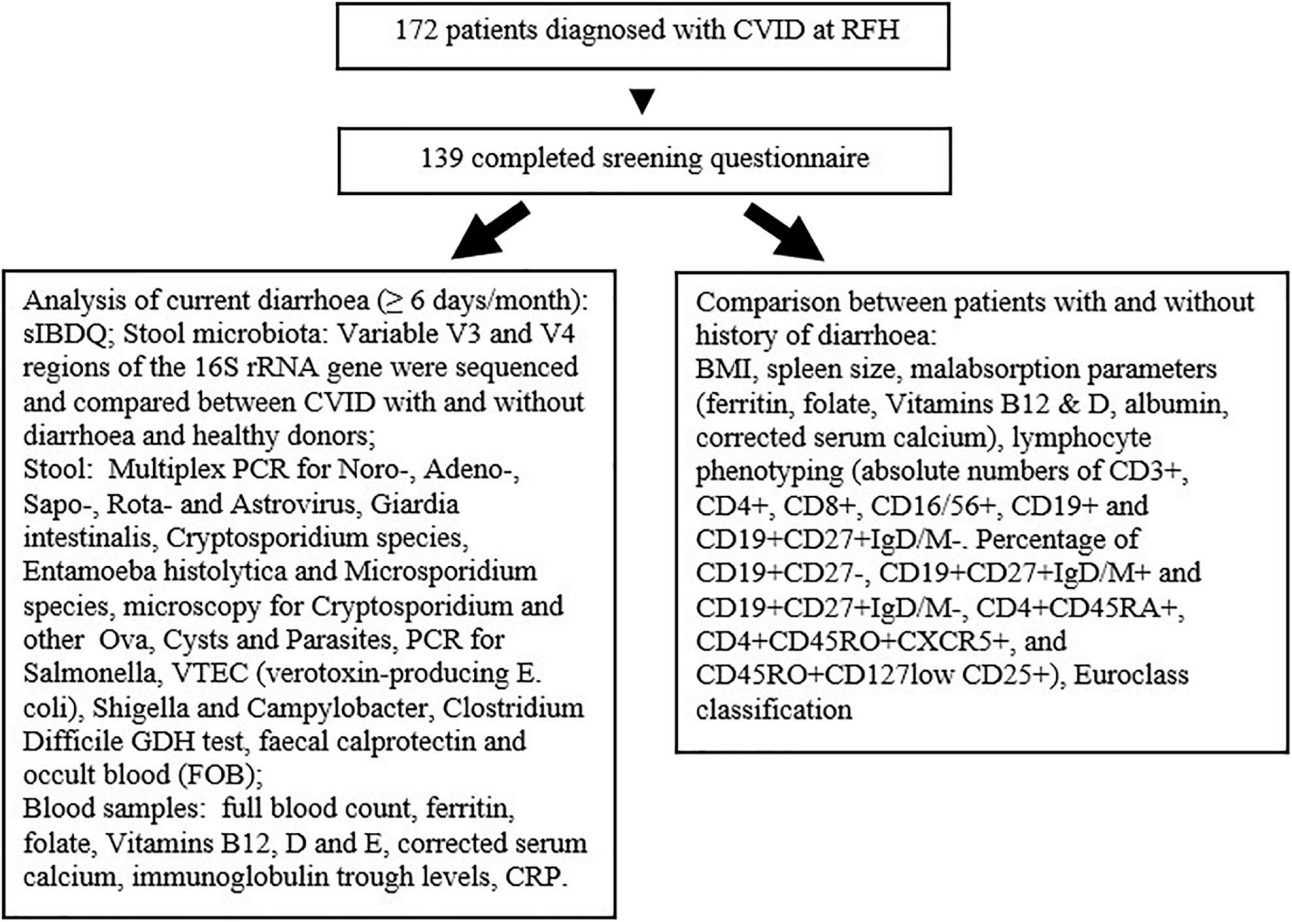
Investigation schema for this study.

We performed a cross-sectional study of adult CVID patients (Figure 1). One hundred thirty-nine out of 172 patients diagnosed with CVID completed a newly created screening questionnaire on past and present gastrointestinal symptoms (see Supplementary Material). CVID had been diagnosed by a consultant immunologist applying standard diagnostic criteria at the time of diagnosis.

Latest body mass index (BMI), spleen size, blood results potentially indicating (mal)absorption, lymphocyte phenotyping, and Euroclass classification were compared between patients with and without a history of diarrhoea according to screening questionnaire.

Patients with significant diarrhoea, defined as loose or liquid stools on ≥6 days/month with no regards to stool frequency had stool analysed for infections, faecal calprotectin, and occult blood (FOB). Blood samples were checked for malabsorption and inflammation and patients completed the sIBDQ. Stool and blood results of one patient who did not complete the screening questionnaire but reported to her doctor constant diarrhoea were included. Four patients who had had episodes of diarrhoea (*n* = 3) or no diarrhoea (*n* = 1) at time of screening questionnaire developed current significant diarrhoea and became eligible for these investigations.

The sIBDQ consists of 10 questions. For each item there is a 7-point Likert scale ranging from 1 for the worst to 7 for the best quality of life in this item. Higher scores represent a better quality of life.

Additional stool samples from patients with current significant diarrhoea were collected and used to analyse participants' gut microbiota and that of a person they lived with. A specific questionnaire [based on the Kieler Fragebogen für Erwachsene (32)] was completed (see Supplementary Material). A CVID control group who did not currently or formerly suffer from diarrhoea was also recruited. Stratec Stool collection tubes with Stool DNA stabilizer (Catalogue #: 1038111200) were used for stool collection. After collection, patients were advised to keep samples in the fridge and post within 24 h. After delivery to the RFH, samples were frozen at −80°C until transported to the CCI Freiburg for sequencing. Variable V3 and V4 regions of the 16S rRNA gene were sequenced using the Illumina protocol on an Illumina MiSeq, referring to the Ribosomal Database Project (33).

In order to compare the microbiota of the three study groups (CVID with and without diarrhoea and healthy household controls) alpha diversity was calculated using Shannon diversity, which weights the numbers of species by their relative evenness (34, 35).

Taxa were classified using operational taxonomic units (OTUs) using the Ribosomal Database Project Classifier (36).

Permutational multivariate analysis of variance [PERMANOVA (37)], implemented in the “adonis” function of the R package “vegan” and used with Bray-Curtis dissimilarity measure, was employed to assess the significance of differences in microbial composition between groups, called beta diversity.

For continuous variables, the Mann-Whitney *U*-test and the Wilcoxon Rank sum test were used. For categorical variables, the Fisher's exact test and Pearson's Chi^2^-test were used. Two-sided *p* ≤ 0.05 were considered significant.

If a question was left unanswered in the screening questionnaire or microbiome questionnaire by a participant, the denominator in our report was changed accordingly.

CVID patients and healthy controls provided written informed consent under study protocols approved by NHS Research Ethics Committees (REC 04/Q0501/119 and 08/H0720/46).

## Results

### Diarrhoea and Other Gastrointestinal Symptoms Are Common in Patients With CVID

**Figure 2:**
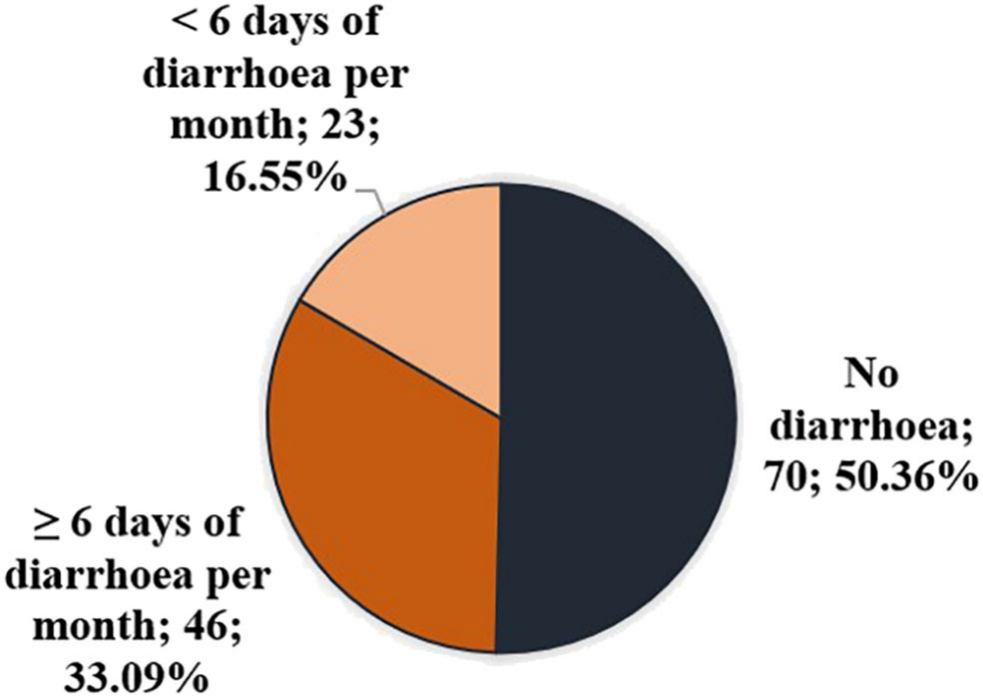
Prevalence of diarrhoea in cohort of CVID patients at time of interview.

One hundred thirty-nine out of 172 patients with the diagnosis of CVID at the RFH were interviewed; demographic details are provided in Table 1. At the time of questioning, 46 of 139 (33.1%) patients had diarrhoea on 6 or more days per month (Figure 2), which we defined as current significant diarrhoea; 69 of 139 (49.6%) patients either had significant diarrhoea at the time of interview or had experienced at least one diarrhoeal episode of 14 days or longer (*n* = 57). For 41 of these patients, episodes persisted for more than 2 months. Diarrhoea in this cohort was mostly a longstanding problem with a median duration of 15.5 years in those patients with current significant diarrhoea. A Bristol Stool Chart Type of 6 or 7 was assigned by 47 out of 63 patients with a history of diarrhoea who answered this question. The gender ratio in the overall cohort was 0.65 compared to 0.60 in the group of patients with a history of diarrhoea, *p* = 0.79.

**Table 1:** Descriptive details of interviewed cohort of CVID patients.

Gender *n* = 139	55 Male	84 Female	Ratio: 0.65
Age at time of interview [years], *n* = 139	Range: 20–84	Mean 49.58	Median 52
Age at diagnosis of CVID [years], *n* = 130	Range: 2–69	Mean 33.95	Median 34
Gender diarrhoea patients, *n* = 69	26 Male	43 Female	Ratio: 0.60
Age at onset of significant diarrhoea [years], *n* = 61	Range: 0–71	Mean 31.91	Median 24
Years of significant diarrhoea, *n* = 61	Range: 0–70	Mean 16.29	Median 15.5


Thirty-eight out of 64 patients with past or present diarrhoea (59.4%) reported at least one of four selected symptoms suggestive of IBD (38, 39). Most frequently this was diarrhoea during the night (*n* = 29), followed by weight loss (*n* = 18). These symptoms were reported by 71.8% of women compared to 40% of men. Only a minority of these patients had chronic infection (see below).

Abdominal pain (32 out of 65) and urgency to defecate (24 out of 68 patients) were also common symptoms. Seventy-one out of 132 CVID patients (53.8%) admitted frequently suffering from bloating, epigastric pain, belching and/or vomiting. Belching and epigastric pain appeared in 13.6% and 12.9% of patients (18 and 17 patients, respectively) and 46.2% (61 patients) frequently suffered from bloating and distension. Significantly more diarrhoea than non-diarrhoea patients frequently had at least one of the additional symptoms listed above (58.0 vs. 39.7%, *p* = 0.039).

### CVID Patients With Diarrhoea Have Lower naïve CD4+ T Cell Counts

Spleen-size, lymphocyte phenotyping, Euroclass classification, BMI, and malabsorption were compared between diarrhoea and non-diarrhoea patients.

Recent spleen measurements were available for 123 patients. Thirty-nine out of 123 patients (31.7%) had splenomegaly (≥14 cm); proportions were equal in the group with and without diarrhoea.

**Figure 3:**
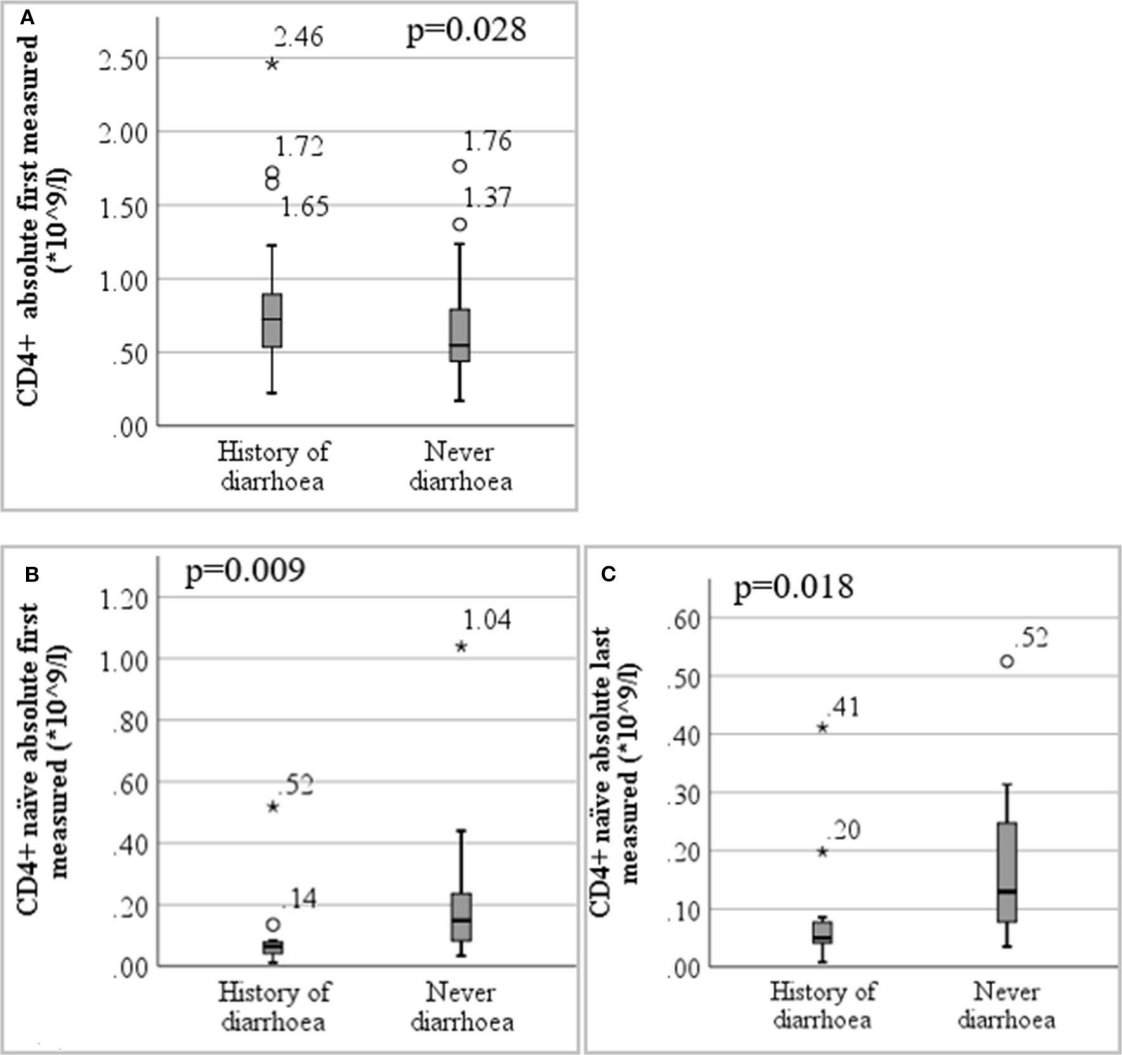
Boxplot of lymphocyte immunophenotyping results detailing **(A)** first measured CD4+ T-lymphocytes in patients with and without diarrhoea (*n* = 138) **(B)** first measured CD4+ naïve T-lymphocytes in patients with and without diarrhoea (*n* = 28) **(C)** last measured CD4+ naïve T-lymphocytes in patients with and without diarrhoea (*n* = 28). *are extreme outlier values (more than three interquartile ranges from the 25^th^ or 75^th^ percentile accordingly).

The first ever measured total CD4+ lymphocyte count in peripheral blood was higher in diarrhoea patients (*p* = 0.028; *n* = 138), but there were significantly lower numbers of (first and last measured) naïve CD4+ lymphocytes in patients with diarrhoea (*p* = 0.009, *n* = 28 and *p* = 0.018, *n* = 28). This is depicted in Figure 3. We did not find low numbers of memory B-cells in blood to be associated with chronic diarrhoea (*p* = 0.266 for comparison of first, *p* = 0.374 for last measured memory B-cells, *N* = 89). There was also no significant difference between the groups in the other measured or calculated lymphocyte numbers (CD3+ T-cells, CD8+ T-cells, CD16/56+ NK cells, CD19+ B cells, switched memory B-cells, naïve B-cells, IgM+ memory B-cells, CD4+ memory T-cells, and T-regulatory cells) (see Supplementary Material).

The Euroclass classification^1^ was available for 131 patients: 78 were classified as smB+, 14 as B-, 32 as smB-Tr^norm^ and seven as smB-Tr^hi^. SmB+ occurred more often in patients without diarrhoea (67.65 vs. 50.79%) but this difference was not significant.

### CVID Patients With Diarrhoea Have Lower Body Mass Index and More Commonly Have Historical Indicators of Malabsorption

The BMI was significantly lower in the group of patients with past or present diarrhoea (median 23.7 in diarrhoea patients vs. 26 in non-diarrhoea patients, *p* = 0.005, *n* = 128). Out of seven underweight patients, five had current significant diarrhoea and two had had diarrhoea episodes of several months' length.

The departmental database was searched for the lowest available measurements of ferritin, folate, Vitamin B12, albumin, and corrected serum calcium as indicators of malabsorption. Malabsorption was significantly more common in patients with diarrhoea than without (40 out of 69 vs. 25 out of 70, *p* = 0.011).

IgA levels in both groups were similar with no significant difference observed (see Supplementary Material).

### Patients With Current Significant Diarrhoea Commonly Have Evidence of Gastrointestinal Inflammation and Malabsorption but Active Infection Is Relatively Rare

Thirty-five patients with current significant diarrhoea supplied stool samples for analysis within 7 months from the interview. Of these, nine had presumed current gastrointestinal infection. For 21 patients, we were able to perform all planned tests.

Four out of 30 patients had norovirus detected, one of these was also positive for adenovirus by PCR. One further patient was negative for norovirus in this study but was later on found to be positive. One patient had CMV inclusion bodies in an ileal biopsy 2 years prior to this study, but was negative for all infectious agents tested in this study (which did not include CMV). One patient was positive by PCR for sapovirus. One of 26 patients was positive for Clostridium difficile GDH, but not for its toxin (therefore not considered as a current infection), one of 29 patients tested positive for *Giardia lamblia* by both PCR and microscopy. One patient had a positive *Campylobacter* stool PCR although stool culture was negative.

Calprotectin was elevated ≥60 μg/g in 13 out of 29 patients, and in 9 out of 21 when excluding patients with known gastrointestinal infections.

One of 23 tested patients was positive for FOB; this was the patient who had earlier been CMV-positive in a biopsy. Two patients had raised α1-antitrypsin levels in their stool (>0.47 mg/g), potentially suggesting protein losing enteropathy. One of these had norovirus, the other one did not have current test results for gastrointestinal viruses.

Blood from 43 patients was analysed in the laboratory within 6 months from the interview. It was possible to obtain all measurements from 30 patients. Five patients were anaemic, two of these with infection (*Giardia*, norovirus). Four of 41 patients were deficient in folate of whom two were due to infection (norovirus and CMV). Five of 40 were deficient in calcium (three due to infection: CMV, norovirus, and *Giardia*), two of 29 were deficient in vitamin E (both had norovirus). Two had IgG serum trough levels <5 g/l, two of 40 were deficient in vitamin B12, four of 41 in vitamin D and one of 43 in albumin (all not due to infection). This amounts to a total of nine of 43 patients (20.93%) presenting with current laboratory signs of malabsorption (excluding vitamin D, IgG trough-level, and anaemia). Of these nine, four patients had an infection (norovirus twice, CMV, and *Giardia*).

### CVID Patients With Diarrhoea Have an Impaired Quality of Life

Twenty-six patients with current significant diarrhoea completed the short Inflammatory Bowel Disease Questionnaire. Most questions ask about the impact of bowel problems during the last 2 weeks and higher scores represent a better quality of life. The mean score per patient across all 10 questions ranged from 3 to 6.4, the median was at 4.74.

**Figure 4:**
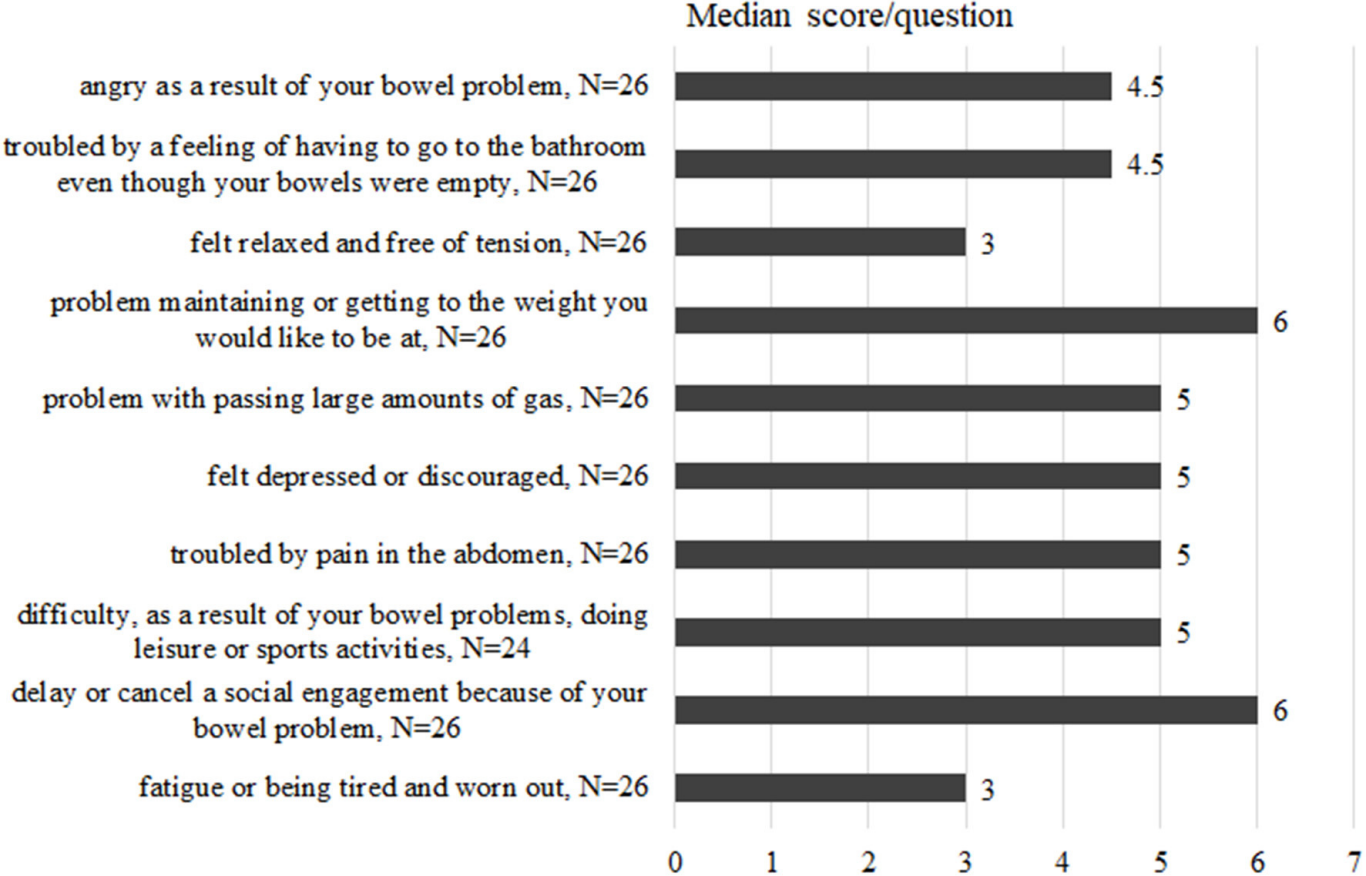
Replies from the sIBDQ completed by 26 CVID patients with current diarrhoea. Questions ask about the impact of bowel problems and quality of life during the last 2 weeks. For each item there is a 7-point Likert scale ranging from 1 for the worst to 7 for the best quality of life in this item. Higher scores represent a better quality of life. Medians for each reply are given in this bar-chart.

Two questions were answered with remarkably low scores, indicating substantially impaired quality of life, as depicted in Figure 4. Those were the questions “How often has the feeling of fatigue or of being tired and worn out been a problem for you during the last 2 weeks?” and “How often during the last 2 weeks have you felt relaxed and free of tension. Notably, these questions are not necessarily linked to the presence of abdominal symptoms. However, very bowel-specific questions, such as “How much of the time during the last 2 weeks have you been troubled by a feeling of having to go to the bathroom even though your bowels were empty?” also received low scores from many patients, and 13 patients replied “some of the time.” This was also the case for the question: “How much of the time during the last 2 weeks have you felt angry as a result of your bowel problem?”. Here, half of the patients assigned replies in the range of 1–4 and said this had been the case “all of the time” (*n* = 1), “Most of the time” (*n* = 3), “A good bit of the time” (*n* = 1), and “Some of the time” (*n* = 8).

### Characteristics of Microbiota Subcohort

Stool samples from 15 CVID patients with diarrhoea were collected. Of these, 12 patients also supplied a sample from a healthy household control. Additionally, 15 CVID patients without diarrhoea provided a sample. The microbiome questionnaire (see Supplementary Material) was answered within a mean of 2.49 days (min. 0, max. 52).

The mean age of participants was 57.21 years at time of sampling (min. 20, max. 81, median 60). There were significantly more females in the “CVID with diarrhoea” group, and more male than female participants in the two other groups.

Fifteen of 30 CVID patients included in the study stated they had been taking systemic antibiotics during the last 6 weeks before sampling. Four CVID patients with and two without diarrhoea were taking systemic glucocorticoids at the time of sampling. Patient characteristics are displayed in Table 2.

**Table 2:** Abdominal pain, stool characteristics, and intake of antibiotics during the last 6 weeks.

	**CVID + diarrhoea**	**CVID no diarrhoea**	**Household controls**	**Significance**
Abdominal pain present “none of the time”	3/15	9/13	9/11	*p* = 0.003
≥4 h on toilet/week	3/15	1/14	0/12	*p* = 0.203
≥3 bowel movements during last 24 h	10/14	1/14	1/12	*p* < 0.001
Bowel movement during last night	4/15	3/14	2/11	*p* = 0.871
≥15 bowel movements during last week	9/15	1/13	1/12	*p* = 0.002
≥8 unformed stools/week	11/15	1/14	1/11	*p* < 0.001
Urgency during last 24 h	10/14	3/14	1/12	*p* = 0.001
Feeling “excellent” during last 2 weeks	1/13	8/14	9/11	*p* = 0.001
Bristol Stool Chart usual type 5–7	9/15	1/14	2/12	*p* = 0.004
Intake of systemic antibiotics during last 6 weeks	9/14	6/14	0/12	*p* = 0.004

*Patients with intake of antibiotics were not excluded*.

### CVID Patients With Diarrhoea Have an Altered Microbiome

The colonic microbiota of CVID patients with diarrhoea differed significantly from that of CVID patients without diarrhoea in beta diversity [*p*-value 0.05 PERMANOVA (37) on Bray-Curtis distance].

**Figure 5:**
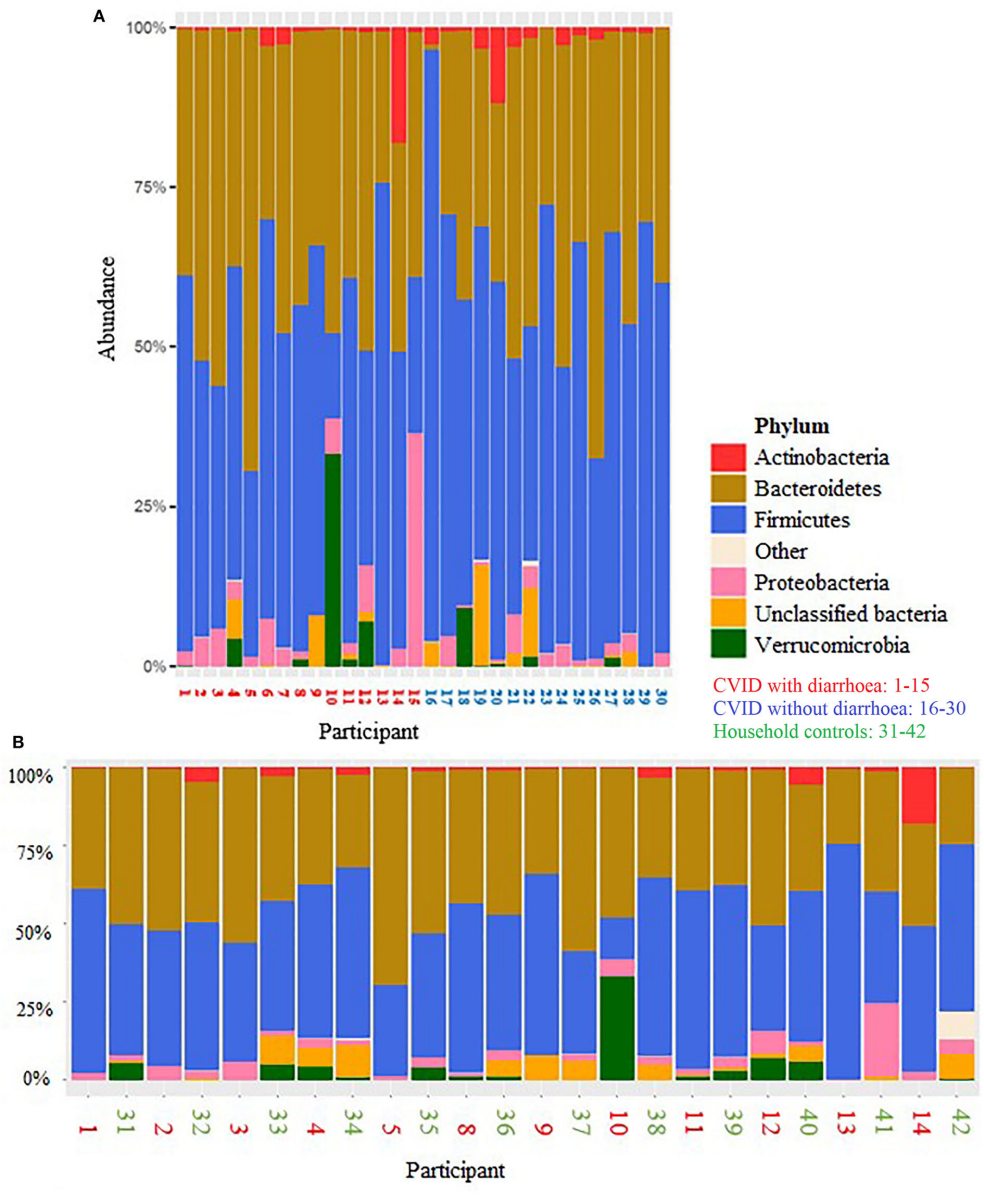
Distribution of taxa at phylum level for **(A)** CVID patients with diarrhoea (red numbers) and CVID patients without diarrhoea (blue) and **(B)** CVID patients with diarrhoea (red) and their healthy household controls (neighbouring in green).

Looking at the two vertical bar charts (Figure 5) that demonstrate the distribution of taxa at phylum level, alterations in two patients with diarrhoea and norovirus are striking. There is an expansion of Verrucomicrobia in patient No. 10 and an expansion of Proteobacteria in patient No. 15.

One further patient was assumed to have been norovirus positive [No. 2, infection interval estimated by calculating divergence and ancestor dates (40)], one had very rare CMV inclusions in an ileal histopathological sample 2 years earlier (No. 6). One was positive for sapovirus in a current stool-PCR (No. 4) and No. 9 was positive for *Giardia lamblia*.

The colonic microbiota of CVID patients with diarrhoea did not differ significantly from that of their healthy household controls in beta diversity, *p* = 0.062, PERMANOVA.

**Figure 6:**
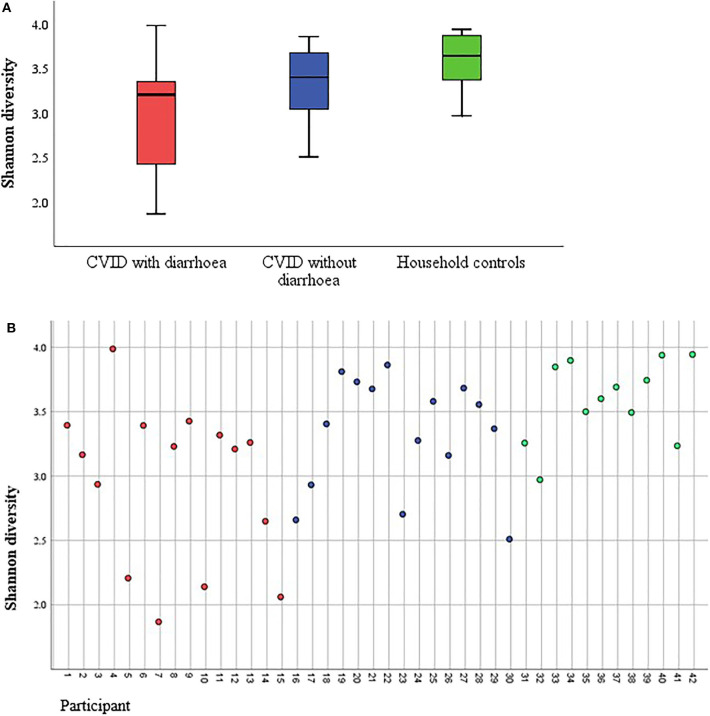
**(A)** Boxplot of stool microbiota alpha diversity in CVID patients with and without diarrhoea, and household controls without immune deficiency. Alpha diversity depicted with Shannon diversity. **(B)** Alpha diversity presented as Shannon diversity for every participant. Red, CVID with diarrhoea; blue, CVID without diarrhoea; green, household controls. Three of the patients with diarrhoea were assumed to have been norovirus positive (No. 2, 10, and 15). No. 4 was sapovirus positive and No. 9 was positive for Giardia lamblia. No. 6 had CMV-inclusions in a previous ileal biopsy.

However, alpha diversity (Figure 6) was significantly lower in CVID patients compared to healthy controls (*p* = 0.009 by Shannon diversity) and specifically in CVID patients with diarrhoea compared to healthy controls (*p* = 0.003). These differences persist when omitting the six participants with assumed infection (CVID *n* = 24 vs. healthy controls *n* = 12, *p* = 0.001; CVID with diarrhoea (*n* = 9) vs. healthy controls (*n* = 12), *p* = 0.02; CVID without diarrhoea (*n* = 15) vs. healthy controls (*n* = 12, *p* = 0.097).

There was no significant difference in alpha diversity between CVID patients with and without diarrhoea (*p* = 0.065) when looking at all participants. However, omitting participants with infection, there was a significant difference in alpha diversity between CVID patients with diarrhoea (*n* = 9) and CVID patients without diarrhoea (*n* = 15), *p* = 0.04.

There was no difference in Shannon diversity between participants with (*n* = 15) and without (*n* = 24) use of antibiotics during the last 6 weeks, *p* = 0.083, nor when only comparing CVID patients with (*n* = 15) and without (*n* = 13) antibiotics during the last 6 weeks, *p* = 0.420.

## Discussion

This study is the largest study to assess longstanding and recurrent diarrhoea in CVID patients by interview. At 33%, the prevalence of longstanding diarrhoea was expectedly high (6–8); in many cases, this was accompanied by abdominal pain, belching, bloating, urgency, or suggestive symptoms of inflammatory bowel disease, although the prevalence of abdominal pain and urgency were not as high as expected.

With its high prevalence, diarrhoea must be seen as a possible warning sign of primary immunodeficiency. This was implemented into the current German guideline for diagnosing PID (41), as suggested by Baumann et al. (42).

A minority of our patients with symptomatic diarrhoea tested positive for infections. Four patients were positive for norovirus at the time of this study. One further patient tested positive after the end of this study, symptomatic with diarrhoea at the time of this study. Woodward et al. (12) suggested norovirus to be causative of the coeliac-like enteropathy observed in CVID. Our study does not look at histology, but all our patients with norovirus infection had villous atrophy on thorough assessment (40). We have recently described our patients with norovirus infection in detail (40).

Sapovirus infection has not been previously described as causative for chronic diarrhoea in CVID patients. Its occurrence has been recorded in renal transplant recipients though (43) and hence it is possible that sapovirus has a pathogenic role in chronic diarrhoea in CVID patients. However, to date we have not seen any infections last more than a period of several weeks. One patient had a positive PCR for *Campylobacter*, although stool-culture was negative. Chronic infection with *Campylobacter* is well-recognised in CVID (8, 15). A further patient tested positive for *Giardia lamblia*, again a commonly described gastrointestinal pathogen (8, 10, 44).

One patient was assumed to have had CMV colitis, as this patient (No. 22) had CMV inclusion bodies in an ileal biopsy, along with ulcers. The patient was not treated with antiviral drugs. There are case reports where a positive effect of virostatic therapy on patients' symptoms has been described (45, 46).

However, most patients did not have clear evidence of infection explaining their symptoms. “CVID enteropathy” has often been classified as an autoimmune phenomenon and grouped with auto-immune phenomena such as autoimmune haemolytic anaemia in studies. So far, no clear autoimmune aetiopathogenesis has been confirmed as causative for CVID enteropathy. Hence, although non-infectious diarrhoea in CVID may have an autoinflammatory origin, it should not be called autoimmune.

Berron-Ruiz et al. found low numbers of memory B-cells to be associated with chronic diarrhoea (47). Our data do not confirm this hypothesis. Diarrhoea correlated with a significantly altered T-lymphocyte population, showing elevated CD4+ lymphocyte numbers, but lower proportions of CD4+naïve lymphocytes. A decrease in naïve CD4+ cells has been shown to be strongly associated with the clinical severity of CVID (48).

Immune dysregulation has been suggested to be associated with enteropathy by Mannon et al., who showed an excess of T-helper (Th)-1 cytokine secretion by lamina propria mononuclear cells in CVID patients with gastrointestinal (GI) symptoms associated with malabsorption, differing from that seen in Crohn's disease (49). Persistent activation of the tumor necrosis factor-system has been demonstrated in subgroups of patients (50). These findings illustrate the role of T-cells and cytokines in CVID-enteropathy and suggest it may be an important aspect.

Regardless of the aetiology of diarrhoea, our sIBDQ results revealed a poor bowel-related quality of life with a median score of 4.74. Voiosu et al. demonstrated that patients with active IBD reached a mean sIBDQ score of 4.8, compared to 5.8 in the group with no endoscopic activity (51). Irvine et al. (18) showed similar results. Quality of life in our CVID patients suffering from diarrhoea is hence as low as in patients with active IBD. The two questions with the lowest scores in our cohort asked for a feeling of fatigue and on having felt relaxed and free of tension during the last 2 weeks. These results signify that recreation and recovery are impeded in this group of patients.

Diarrhoea correlated with low BMI and malabsorption. Previous studies have similarly shown malabsorption in CVID patients with chronic/recurrent diarrhoea (7, 52) and a lower BMI in patients with GI symptoms (49) or villous atrophy (53). This emphasizes the need for suitable therapies for both infection and non-infection associated diarrhoea.

A seminal study performed by Jorgensen et al. examined the diversity of gut microbiota of CVID patients including CVID patients with enteropathy (21). In contrast to this first study, we also included patients with chronic infection and with recent antibiotic usage into our analysis, reflecting “real-world” CVID-associated diarrhoea. Jorgensen et al. (21) found that CVID patients with autoimmune complications (as a group including enteropathy) had a lower alpha diversity than CVID patients without autoimmunity. Also, CVID patients in general had a lower alpha diversity than healthy controls (21). This was also observed by Fiedorova et al. (25). In our study, a significant difference in alpha diversity could be shown between CVID patients and healthy donors as well as between CVID patients with non-infectious diarrhoea and those without diarrhoea. We also observed a significant difference in beta diversity between CVID patients with and without diarrhoea, regardless of infection status. This means that the composition of microbiota was different between these groups. However, this should not be over-interpreted as the household control groups' beta diversity was not significantly different from that of the group of CVID patients with diarrhoea.

It is known that the repeated intake of antibiotics can alter the gut microbiota, beyond clinical recovery (29). Jorgensen et al. (22) demonstrated that Rifaximin provokes a reversible reduction in alpha diversity and an increase of beta diversity in CVID patients without changing the composition of dysbiotic bacteria according to their “dysbiosis index.” CVID patients receive considerable amounts of antibiotics due to severe and recurrent infections throughout their lives. Our data did not show a clear impact of antibiotics but the numbers in each group were relatively small and inter-individual differences in microbiota can render comparisons within small cohorts challenging.

Absence of B-cells was shown to have an impact on the microbiota and on malabsorption in mice (54, 55). It has thus been suggested that an altered gut microbiome might play an important role in the pathogenesis of CVID enteropathy (54, 56). Our results, indicating an altered microbiome in patients with non-infectious diarrhoea, would support this.

Notably, a generally reduced microbial diversity in inactive ulcerative colitis (57) as well as in active IBD (58) has been shown. It would therefore be interesting to know if the microbial diversity remains altered in CVID patients who cease to suffer from diarrhoea. This would suggest that “CVID enteropathy” patients retain an altered microbial diversity as a risk for relapse, but also that changing the microbiotic profile to reduce symptoms might be unnecessary as patients may go into remission despite their altered microbial diversity [similar to the UC patients investigated by Martinez et al. (57)]. At this point it is not clear whether an altered microbiome is the initial driver of enteropathy or whether CVID-related pathological changes in gut function lead directly to the altered microbiome. Longitudinal studies on the microbiome of CVID patients with and without gastrointestinal disease would help to determine this.

We also observed marked changes in the dominant phyla of two patients with norovirus and diarrhoea: one had an expansion of Verrucomicrobia, the other of Proteobacteria. This was similarly observed by Lees et al. (59) in children. Proteobacteria were also more abundant in a study on norovirus infected mice (60) and in some norovirus infected humans (61). There are no previous studies for comparison on the microbiome of CVID patients with infectious diarrhoea.

Our study does have limitations. The screening questionnaire was designed to be a sensitive tool to detect all patients who had current or past diarrhoea even at a relatively modest level. There may have been a response bias, as before patients were contacted, clinic letters and databases were searched for possible GI symptoms and a higher emphasis was made to recruit these patients. Also, patients with abdominal symptoms might have been more motivated to complete the questionnaire.

A second bias influencing the results could be a recall bias. Patients with past diarrhoea might remember their symptoms more or less severely than they actually were. Severity of non-abdominal symptoms might influence how important patients find their GI symptoms.

Patients left out occasional questions in the questionnaires and it was not possible to obtain results for all tests (especially those collected retrospectively), meaning that the datasets were inevitably incomplete.

Stool tests were not always performed on days with diarrhoea. It is unclear whether pathogens can more often be found on days when there are symptoms if diarrhoea is intermittent. In three cases, infection status was not based on current test results but on earlier histology (1 case) and on an estimated infection interval (2 cases).

Overall, our results support the hypothesis that CVID patients commonly suffer bowel disease (16), sometimes associated with infection. CVID patients with diarrhoea of any aetiology have an altered microbiome. The impact of diarrhoea on CVID patients' quality of life is high. It is important to first establish a generally accepted definition of “CVID enteropathy” which we propose should include all CVID patients with longstanding diarrhoea or proven mucosal abnormality on biopsy not attributable to infection. This could be further classified according to the presence or absence of gastrointestinal inflammation, malabsorption, and histological abnormalities. Subsequently, trials of effective treatment to relieve affected patients are needed.

## Data Availability Statement

The original data on 16S rRNA sequencing presented in the study are publicly available. This data can be found here: https://doi.org/10.5061/dryad.3j9kd51fz.

## Ethics Statement

The studies involving human participants were reviewed and approved by National Research Ethics Service Committee London-Hampstead and National Research Ethics Service North West London. The patients/participants provided their written informed consent to participate in this study.

## Author Contributions

This study was conceptualised by BG, DL, and CS. Questionnaires were created by BG, DL, and CS, the questionnaire for analysis of microbiota was based on Kieler Fragebogen für Erwachsene, Studie zur Rolle des Mikrobioms, Erwachsene, Version 1.2, 1st August 2014. Access to clinical data was granted by the Immunology Department, Royal Free Hospital London with support by SB. SW and AS took care of patients' consents and organising appointments. Gaining of clinical data, questioning of patients, gathering samples, and analysis of data was primarily executed by CS. Additionally data was extracted by DG, SA, and FM. Sequencing of stool samples was executed by CN, analysis through AB and MP. Advise for statistical analysis was given by VS. All authors contributed to the article and approved the submitted version.

## Conflict of Interest

The authors declare that the research was conducted in the absence of any commercial or financial relationships that could be construed as a potential conflict of interest.

## Footnotes

^1^B– = ≤ 1% B-cells; smB = >1% B-cells; smB+ = >2% switched memory B-cells; smB– = ≤ 2% switched memory B-cells; smB-Tr^hi^ = ≥9% transitional B-cells; smB-Tr^norm^ = <9% transitional B-cells.

## Abbreviations

BMI: body mass index
BSC: Bristol Stool Chart
CMV: cytomegalovirus
CVID: common variable immunodeficiency
GI: gastrointestinal
IBD: inflammatory bowel disease
IvIg: intravenous immunoglobulins
No.: number
PID: primary immunodeficiency
RFH: Royal Free Hospital London
sIBDQ: short Inflammatory Bowel Disease Questionnaire.
