# Supplementary material for: Altered Microbiota, Impaired Quality of Life, Malabsorption, Infection, and Inflammation in CVID Patients With Diarrhoea
Source: Front Immunol. 2020 Jul 31;11:1654. doi: 10.3389/fimmu.2020.01654 (PMC7412961; doi:10.3389/fimmu.2020.01654)
Supplement: Supplementary file 1 [file Data_Sheet_1.docx]

Altered microbiota, impaired quality of life, malabsorption, infection, and inflammation in CVID patients with diarrhoea

Supplementary material

# Questionnaire diarrhoea

Questionnaire on abdominal symptoms for CVID patients

Dear patient,

Diarrhoea and other abdominal symptoms often occur in CVID patients. In order to get a better insight into the symptoms and circumstances, we kindly ask you to complete the following questionnaire. If sections do not apply to you there are instructions to skip them.

Thank you very much for your contribution!

NAME: _________________________________________ DATE: _____________

1. Current status of diarrhoea
   1.1 **Is diarrhoea a problem that you often get?**  ☐YES ☐NO
   If you ticked “YES” here, please go on to section B.
   **1.2 Do you have diarrhoea today** (at least 3 loose/liquid stools/day)? ☐YES ☐NO
   If you ticked “NO” here, please go on to section B.
   1.3 Current frequency of **bowel movements/day**:
   1.4 **For how many days** have you been having diarrhoea?
   1.5 How acute was the **onset**? ☐Explosive, ☐gradual, ☐I always have diarrhoea
   1.6 Does diarrhoea stop when **fasting**? ☐YES ☐NO
   1.7 Do you have diarrhoea during the **night**? ☐YES ☐NO

Stool characteristics:
2.1 What **colour** does it have today? ☐Brown, ☐pale, ☐yellow, ☐green, ☐black
2.2 Please assign a Bristol Stool chart type to your stool. You will find the chart on the back of this questionnaire. ☐1, ☐2, ☐3, ☐4, ☐5, ☐6, ☐7
2.3 Is there ☐blood, ☐mucus or ☐pus in it? ☐No
2.4 Is the stool **floating** in toilet? ☐YES ☐NO

Circumstances:
3.1 Was there a **trigger** for diarrhoea to start? ☐YES: _____________________ ☐NO
3.2 Have you suffered from **abdominal pain** during this diarrhoea episode? ☐YES ☐NO
3.3 Have you unintentionally **lost weight** during this diarrhoea episode?
☐YES: lb or kg ☐NO
3.4 Have you had **raised temperature**? ☐YES: °C ☐NO
3.5 Have you recently **changed your diet** (previous to this episode)? ☐YES ☐NO
If yes: How? ____________________________________________When? _______
3.6 Have you had **antibiotic treatment** within two months previous to this episode? ☐YES ☐NO If yes: With which antibiotic drug? ___________________
3.7 Have you recently, previous to this episode, received **any other new medication** that you did not use to take before? ☐YES ☐NO
If yes: Which? _____________________________________ When?____________
3.8 Please tick if you take any of these drugs regularly: ☐**PPI (I.e. Omeprazole, Lanzoparzole), ☐Laxatives,☐NSAIDs** (I.e. aspirin, ibuprofen), **☐Digoxin**?
3.9 Have you travelled/lived outside the UK during the last 8 weeks?
☐YES, to _________________________________________________ ☐NO
3.10 Have you ever travelled to the tropics? ☐YES ☐NO When? ______________

Therapy:
4.1 Have you already received/taken **medication** to improve **this episode**? ☐YES NO If yes: Which? ☐Steroids (which? : _______________________), ☐antibiotics

(which?: ______________________________), ☐Loperamide, ☐codeine phosphate,

☐ probiotics, ☐Other: ________________________?
If you take any of the above regularly, please encircle.
Did it **help improve** your symptoms? ☐YES ☐NO ☐slight improvement
4.2 Have you **changed your diet** to improve your symptoms? ☐YES ☐NO
If yes: How? ☐Lactose free diet, ☐fructose reduced diet, ☐gluten free diet, ☐vegan diet
Did this **help improve** your symptoms? ☐YES ☐NO ☐slight improvement

1. History of diarrhoea

5.1 How many days of a normal month do you have diarrhoea (at least 3 loose/liquid stools/day)? days.
5.2 Have you **ever had continuous diarrhoea** (at least 3 loose/liquid stools/day) **for at least 14 days in a row**? ☐YES ☐NO
**If yes, how many weeks in a row** did/do you suffer from diarrhoea during one diarrhoea episode? ☐2-4 weeks, ☐4-8 weeks, ☐several months,
☐ Variable length, ☐I always have diarrhoea
**If yes, how many episodes** of 14 days or longer have you had during **your life**? Tick: ☐1-3, ☐more than 3, ☐I have diarrhoea most of the time.

Please go on to section E if you don’t usually get diarrhoea and have never had a 14day diarrhoea episode.

5.3 When did diarrhoea start to become a problem?
Age of onset: or year of onset:

Stool characteristics:
How many **bowel movements** do/did you have every day when having diarrhoea? Bowel movements/day
6.1 What **colour** did/does it have? ☐Brown, ☐pale, ☐yellow, ☐green, ☐black
6.2 Please assign a Bristol Stool chart type to your stool. You will find the chart on the back of this questionnaire. ☐1, ☐2, ☐3, ☐4, ☐5, ☐6, ☐7
6.3 Was/is there ☐blood, ☐mucus or ☐pus in it? ☐No
6.4 Stool **floating** in toilet? ☐YES ☐NO

Circumstances:
7.1 Was/is there a **trigger** for diarrhoea to start? ☐YES:____________________ ☐NO
7.2 Had you travelled/lived outside the UK prior to the start of diarrhea (e.g. the tropics)? ☐YES ☐NO Where? ________________________
7.3 Did/do you have **raised temperature in times of diarrhoea**? ☐YES: ____°C ☐NO
7.4 Do you unintentionally **lose weight** during diarrhoea episodes?
 ☐YES: lb or kg ☐NO
7.5 Did/do you have diarrhoea also during the **night**? ☐YES ☐NO

Therapy:
8.1 Did/does diarrhoea get better when **fasting**? ☐YES ☐NO
8.2 Did/do you receive/take any **treatment**? ☐YES ☐NO
**If so, which?** ☐Steroids (which?: _______________________________), ☐antibiotics (which?: _______________________________________), ☐loperamide, ☐codeine phosphate, ☐probiotics, ☐other: _________________________________________
If you take any of the above regularly, please encircle.
Did the drugs **help improve** your symptoms? ☐YES ☐NO ☐slight improvement
Was the medication prescribed at RFH? ☐YES ☐NO
**For how long** did you have to take drugs? ____________________________
**When** was that? ____________________________
8.3 Have you tried a **change of diet** to improve your symptoms? ☐NO, I haven’t
☐YES: ☐Lactose free diet, ☐fructose reduced diet, ☐gluten free diet, ☐Vegan.
Did it help? ☐YES ☐NO ☐slight improvement

1. Pain and urgency
   9.1 Do you have abdominal pain in times of diarrhoea?☐YES ☐NO

***If “NO”, please go on to D.***If yes: Where? _______________________________________________
9.2 Pain relieved when opening bowel? ☐YES ☐NO
9.3 Pain worse when you have diarrhoea? ☐YES ☐NO
9.4 Urgency to go to the toilet? ☐YES ☐NO
9.5 Abdominal bloating/distension? ☐YES ☐NO
9.6 Mucous from the rectum? ☐YES ☐NO
9.7 Worsening after food? ☐YES ☐NO
9.8 Symptoms ≥ 6 months? ☐YES ☐NO
9.10 A feeling that you have not emptied your bowels completely after going to the toilet? ☐YES ☐NO

Diarrhoea in your close environment
10.1 Does the person you live or work with suffer from diarrhoea either currently or chronically? ☐YES ☐NO
If yes: Is it ☐chronic (>14days in a row) or ☐acute diarrhoea?
If yes: what is the diagnosis? ___________________________________________
If yes: Who is it? ☐Parent, ☐partner, ☐child, ☐colleague, ☐sibling, ☐flat-mate, ☐close friend

Medical history:
11.1 Please tick if you have been diagnosed with **any of the following:** ☐Crohn’s disease, ☐ulcerative colitis, ☐coeliac disease, ☐microscopic colitis, ☐diabetes, ☐hyperthyroidism, ☐hypothyroidism, ☐irritable bowel syndrome, ☐tuberculosis, ☐Whipple’s disease, ☐cystic fibrosis, ☐chronic pancreatitis, ☐gastritis, ☐gastric or duodenal ulcers, ☐exocrine pancreatic insufficiency, ☐NONE of them?
11.2 Have you had your **gall bladder removed?** ☐YES ☐NO
11.3 Do you **frequently suffer from** ☐epigastric pain, ☐bloating, ☐belching, ☐vomiting? ☐No
11.4 Do you regularly take supplements such as ☐iron, ☐vitamin B 12, ☐calcium, ☐vitamin D, ☐Folate, ☐other:____________________☐I don’t take any supplements.
11.5 What is your current prophylactic antibiotic substance? _____________________

Endoscopy:
12.1 Have you ever had endoscopy (colonoscopy, gastroscopy, capsule endoscopy) or MRI of the abdomen?
☐NO ☐YES: please specify: ___________________________

Where? __________________________________________

When? _________________________________________

Why? _________________________________________

Findings? _________________________________________

Thank you very much for completing this questionnaire and supporting our research on your condition!

# Questionnaire Microbiome (including sIBDQ: Q53 - 62)

General questions

1. I live…
   ☐Alone
   ☐with my partner/flat-mate
   ☐with my children
   ☐Other:_________________________
2. Do you have any children? (non-adopted) ☐Yes ☐no
3. Do you have any siblings? ☐Yes ☐no
   How many siblings?
   How many half-brothers/sisters?
4. My own birth was... ☐normal (=vaginal) ☐C-section ☐I don’t know
5. Did your mother have gestational diabetes while being pregnant with you?
    ☐Yes ☐no ☐I don’t know
6. Did your mother breast-feed you? ☐Yes ☐no ☐I don’t know
   For how many months?
7. Which of the following applies to you?
   ☐I have never smoked or I have smoked less than 3 months
   ☐I do not smoke anymore now but have smoked more than 3 months before stopping.
   ☐I smoke.
   If you smoke, how many cigarettes/day?
8. Did you go to a nursery as a child? ☐Yes ☐no ☐I don’t know
   If yes: from which age on?
9. Where did you grow up most of the time? (Only one tick please)
   ☐Very large city: (>1,000,000 inhabitants) ☐Large city (100,000 – 1,000,000 inhabitants) ☐Intermediate town (20,000 – 100,000 inhabitants) ☐Small town (5,000 – 20,000 inhabitants) ☐village (<5,000 inhabitants) ☐rural area with scattered houses
10. Did you regularly visit farms during your childhood? ☐Yes ☐no
11. Where do you live now? (Only one tick please)
    ☐Very large city: (>1,000,000 inhabitants) ☐Large city (100,000 – 1,000,000 inhabitants) ☐Intermediate town (20,000 – 100,000 inhabitants) ☐Small town (5,000 – 20,000 inhabitants) ☐village (<5,000 inhabitants) ☐rural area with scattered houses
12. Do you regularly visit farms now? ☐Yes ☐no
13. How many persons lived in the same household as you when you grew up?
    Number (without counting yourself):
14. How many persons live in your current household with you (Do not count
    yourself)?
15. Do you eat fruit and vegetables every day? ☐Yes ☐no
    If yes, please tick the appropriate amount:
    Daily more than 4 servings of fruit and/or vegetables ☐
    Daily more than 2 servings of fruit and/ or vegetables ☐
    One or two servings/day ☐
16. Do you eat meat? ☐Yes ☐no
    If yes, please tick the appropriate amount:
    Daily at least one portion of meat or poultry ☐
    At least four portions of meat or poultry every week ☐
    At least two portions of meat or poultry every week ☐
    Less often than this ☐
17. Do you eat fish? ☐Yes ☐no
    If yes, please tick the appropriate amount:
    Twice every week or more often ☐
    Once every week ☐
    Less often than this ☐
18. Do you eat whole-grain bread, whole-grain rice, muesli, oats or whole grain pasta?
    ☐Yes ☐no
    If yes, please tick the appropriate amount:
    Daily at least one serving of any of these ☐
    At least four servings of any of these every week ☐
    At least two servings of any of these every week ☐
    Less than this ☐
19. Do you eat carbohydrates such as pasta, potatoes or rice? ☐Yes ☐no
    If yes, please tick the appropriate amount:
    One serving every day ☐
    Several servings per day ☐
20. Do you consume dairy products (i.e. milk, yoghurt, cheese etc.)? ☐Yes ☐no
    If yes, please tick the appropriate amount:
    Several servings daily (i.e. one glass of milk, one yoghurt=one serving…) ☐
    One serving/day maximum ☐
    Several servings per week ☐
    Less often than this ☐
    **If NO**, why?
    Because I don’t like dairy products ☐Yes ☐no
    Because I can’t tolerate dairy products ☐Yes ☐no
21. Do you eat sweets? ☐Yes ☐no
    If yes, please tick the appropriate amount:
    Daily at least once ☐
    At least four times every week ☐
    At least twice every week ☐
    Less often than this ☐
22. Do you regularly consume probiotics (ie. Yaccult, Actimel, etc…)? ☐Yes ☐no
23. Do you drink tap water daily? ☐Yes ☐no
24. Do you drink more than 5 drinks of alcohol/day? ☐Yes ☐no
25. Have you been abroad for more than 4 weeks during the last 12 months?
    ☐Yes ☐no if yes: Where? _____________________________________

A short medical history

1. Have you ever been diagnosed with appendicitis? ☐Yes ☐no
   Age at diagnosis (approximate):
2. Has a physician ever told you that you have raised blood lipids? ☐Yes ☐no
3. Are you lactose intolerant? ☐Yes ☐no ☐I don’t know
4. Do you get mouth ulcers three times per year or even more often?
    ☐Yes ☐no
5. Have you ever been diagnosed with an inflammation of your gums (Periodontitis, Gingivitis)? ☐Yes ☐no
6. Have you ever had surgery performed on your bowel? ☐Yes ☐no

If yes, please list in the below table:

| Reason for operation: | Age at that time or year of incident: |
| --- | --- |
|  | Age:  Or year: |
|  | Age:  Or year: |
|  | Age:  Or year: |
|  | Age:  Or year: |
|  | Age:  Or year: |

1. How many courses of antibiotics have you taken during the last 2 years in total?
   ☐None 1-3☐ 4-6☐ More than 6 ☐
2. What is your usual break-through antibiotic? __________________________________
3. What is your usual prophylactic antibiotic substance? ____________________________
4. Have you taken any antibiotic substances during the last 6 months? ☐Yes ☐no
5. Which antibiotic, antiviral or antifungal drugs have you taken during the last 6 weeks (including prophylactic)?

| Antibiotic substance name | Infection that was treated | Number of days that you took this antibiotic | When was that? |
| --- | --- | --- | --- |
|  |  |  |  |
|  |  |  |  |
|  |  |  |  |
|  |  |  |  |

1. Do you take commercial probiotics every day? ☐Yes ☐no
   If yes, which product? ________________________________ Which dose? ________________________
2. Do you have any biological relatives who have been diagnosed with inflammatory bowel disease (IBD) or coeliac disease? ☐Yes ☐no ☐I don’t know
   If yes, please tell us
   Who has it? (I.e. daughter, brother, grandmother from the father’s side etc.)
   Which IBD or coeliac disease? (I.e. Ulcerative colitis, Crohn’s disease, coeliac disease… other).
   At what age was the diagnosis made?

| Who is it? | Which diagnosis? | Age at time of diagnosis or year of diagnosis: |
| --- | --- | --- |
|  |  | Age:  Or year: |
|  |  | Age:  Or year: |
|  |  | Age:  Or year: |

1. This question is about yourself: Have you ever received therapy to treat your bowel symptoms, IBD or coeliac disease? The list includes complementary and alternative therapeutic measures. ☐Yes ☐no
   Which?

| Mesalacine | ☐yes ☐no |
| --- | --- |
| Sulfasalazine | ☐yes ☐no |
| Budesonide | ☐yes ☐no |
| Prednisolone | ☐yes ☐no |
| Antibiotics (i.e. metronidazole, tinidazole) | ☐yes ☐no |
| Azathioprine | ☐yes ☐no |
| 6- Mercaptopurine | ☐yes ☐no |
| Infliximab OR TNF-α-inhibitor | ☐yes ☐no |
| Methotrexate | ☐yes ☐no |
| Cyclosporin | ☐yes ☐no |
| Tacrolimus | ☐yes ☐no |
| Flea seeds (Ispaghula) | ☐yes ☐no |
| Yoga | ☐yes ☐no |
| Acupuncture | ☐yes ☐no |
| TSO (Trichirus suis ovata) | ☐yes ☐no |
| Curcumine | ☐yes ☐no |
| Herbal medicine | ☐yes ☐no |
| Regular intake of probiotics (i.e. Yakult, Actimel, probiotic yoghurt) | ☐yes ☐no |
| Homoeopathic remedies | ☐yes ☐no |
| Other: | ☐yes ☐no |

Questions on your current symptoms and wellbeing

1. During the last 3 months: Did you have abdominal pain or bowel problems on three days/month or more often? ☐Yes ☐no
2. Do you often suffer from constipation? ☐Yes ☐no
3. How many hours did you approximately spend on the toilet during the last 7 days?
   ☐Less than 2 hours ☐2-4 hours ☐4-7 hours ☐7–12 hours
   ☐more than 12 hours
4. How many times did you open your bowel yesterday from getting up in the morning to going to bed at night?
5. How many times did you open your bowel during the last night?
6. How many times do you open your bowel in an average week?
7. Of these how many are soft, liquid or watery?
8. Did you have blood in your stool during the last 24 hours? ☐Yes ☐no
9. How urgently did you have to go to the toilet during the last 24 hours?
   ☐Normal ☐urgent ☐continuous bowel opening but able to control
   ☐Not able to control/ incontinent


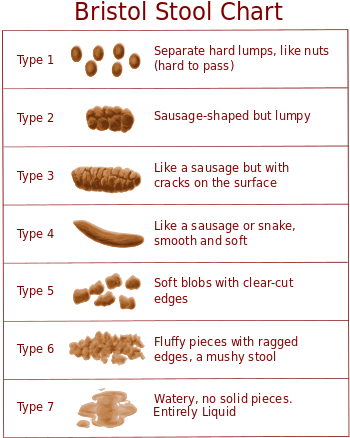


1. Did you have fever during the last 7 days? ☐Yes ☐no
2. How has your general well-being been during the last 7 days?
    ☐Excellent ☐a bit impaired ☐Bad ☐very bad ☐Unbearable
3. If you receive intravenous immune globulins, when was your last infu sion?_____________________
4. Looking at the scale on the left (Bris tol Stool Chart), which type matches your normal stool the most?
    Type _________

Questions on your quality of life

1. How often has the feeling of fatigue or of being tired and worn out been a problem for you during the last 2 weeks?
2. How often during the last 2 weeks have you had to delay or cancel a social engagement because of your bowel problem?
3. How much difficulty have you had, as a result of your bowel problems, doing leisure or sports activities you would have liked to have done during the last 2 weeks?
4. How often during the last 2 weeks have you been troubled by pain in the abdomen?
5. How often during the last 2 weeks have you felt depressed or discouraged?
6. Overall, in the last 2 weeks, how much of a problem have you had with passing large amounts of gas?

1. Overall, in the last 2 weeks, how much a problem have you had maintaining or getting to, the weight you would like to be at?
2. How often during the last 2 weeks have you felt relaxed and free of tension?
3. How much of the time during the last 2 weeks have you been troubled by a feeling of having to go to the bathroom even though your bowels were empty?
4. How much of the time during the last 2 weeks have you felt angry as a result of your bowel problem?

Thank you very much for making this important contribution to our work!
